# Effect of Spray Drying Encapsulation on Nettle Leaf Extract Powder Properties, Polyphenols and Their Bioavailability

**DOI:** 10.3390/foods11182852

**Published:** 2022-09-15

**Authors:** Ena Cegledi, Ivona Elez Garofulić, Zoran Zorić, Marin Roje, Verica Dragović-Uzelac

**Affiliations:** 1 Faculty of Food Technology and Biotechnology, University of Zagreb, Pierottijeva 6, 10000 Zagreb, Croatia; 2 Ruđer Bošković Institute, Biljenička Cesta 54, 10000 Zagreb, Croatia

**Keywords:** spray drying, nettle leaves, maltodextrin, gum arabic, β-cyclodextrin, antioxidant capacity

## Abstract

Nettle (*Urtica dioica* L.) is a plant rich in a health-promoting compounds such as polyphenols, which are sensitive and unstable compounds with low bioavailability, that need to be stabilized and protected from external influences. Therefore, the aim of this study was to examine how the temperature, type of carrier and sample to carrier ratio influence the physicochemical properties and encapsulation and loading capacity of the nettle leaf extract powder and examine the effect of encapsulation on the antioxidant capacity and bioavailability of polyphenols. The process yield ranged from 64.63–87.23%, moisture content from 1.4–7.29%, solubility from 94.76–98.53% and hygroscopicity from 13.35–32.92 g 100 g^−1^. The highest encapsulation (98.67%) and loading (20.28%) capacities were achieved at 160 °C, β-CD:GA (3:1) and sample:carrier ratio of 1:3. Extracts encapsulated at selected conditions showed high antioxidant capacity and distinct polyphenolic profile comprised of 40 different compounds among which cinnamic acids were the most abundant. Moreover, the encapsulation increased the bioavailability of nettle leaf polyphenols, with the highest amount released in the intestinal phase. Thus, the obtained encapsulated extract represents a valuable source of polyphenols and may therefore be an excellent material for application in value-added and health-promoting products.

## 1. Introduction

It is known that in nature there is a large number of plant species with undervalued biological potential that can be processed into various types of products, among which plant extracts certainly occupy an important place [1]. The reason for the popularity and growth of plant extracts is the increasing awareness among consumers about the quality of the food they consume, the presence of bioactive ingredients, and their potential beneficial effects on health [2]. For standardization and stabilization purposes, liquid plant extracts are often processed into powders that can be used as semi-finished or finished products.

Nettle (*Urtica dioica* L.) is one of the medicinal plant species that, due to its chemical composition and content of bioactive components, is an excellent basis for obtaining products with high biological potential. It is a perennial wild plant, known in folk medicine since ancient times, widely distributed and adapted to different climatic zones [3]. It is used both as food and medicine, as all parts of the nettle (leaf, stem, root) are a rich source of antioxidant phenolic compounds, vitamins and minerals. Accordingly, nettle is consumed in the form of tea, stews, soups, juices, etc. [4,5]. Although all parts of nettle contain significant amounts of biologically active molecules and possess medicinal properties, the leaves are the most valuable source [6,7]. The bioactive constituents of nettle leaves, among which phenolic compounds occupy an important place, act as radical scavengers and play an important role in the prevention of cancer, neurodegenerative and cardiovascular diseases, which is one of the reasons for the potential use of nettle leaves in the production of plant extracts, powders, etc. [8,9].

The emphasis on the use of plant products rich in bioactive ingredients that have a positive effect on human health, also increases the need to apply techniques that would lead to the production of foods with high stability and long shelf life. For these reasons, the obtained liquid plant extracts are often transformed into powder form. One of the ways to achieve this form is encapsulation by spray drying, in which liquid or semi-liquid foods are dried in a stream of hot air to produce a powder as the final product. The goal of this process is to quickly and efficiently remove the water from the food and obtain a powder with the desired physicochemical properties [10]. In addition, a physical barrier is created by protecting the unstable active ingredient from external influences (light, moisture, oxygen) [11,12]. Spray drying is an alternative to improve the preservation of the final product. The result is a product with higher stability, better quality, controlled release of biologically active molecules, longer shelf life and lower volume and weight, which facilitates storage, handling and transportation of the product [13,14]. The production of powders with desirable physicochemical properties is highly influenced by the properties of the solution to be dried, the characteristics and parameters of the spray drying equipment, and the appropriate choice of the carrier and its proportion in the mixture [15]. The most commonly used carriers in spray drying are polysaccharides (starch, maltodextrin, gum), proteins (gelatin, casein, soy proteins) and lipids (waxes, glycerides). In the food industry, these are maltodextrin (MD), gum arabic (GA) and β-cyclodextrin (β-CD). They are used due to their wide commercial availability, low cost, high solubility, low viscosity and ability to stabilize the product [16]. Since encapsulants have the potential to be used in a functional product, it is important to monitor their bioavailability as well. The bioavailability of polyphenolic compounds depends significantly on the structure and form in which they are taken into the body, and encapsulation has shown a protective effect on changes in pH and enzymatic activity during the digestive process. In this way, the polyphenols are delivered to a specific part of the digestion and released in a controlled manner [17,18]. From the above, it is clear that in order to obtain products with the best sensory and nutritional properties and higher yields, it is important to first optimize the encapsulation process itself.

According to the authors, there is only one work dealing with spray drying of nettle extract [19]. In that work, the extract was prepared by conventional techniques and the effects of temperature, flow rate, and different concentrations of maltodextrin on the process yield, total polyphenols, encapsulation efficiency, antioxidant activity and morphology of the powder were studied. That work differs from the methodology used in mentioned study. The aim of this study was to encapsulate a nettle leaf extract rich in phenolic compounds and to investigate the influence of temperature (120, 160 and 200 °C), type of carrier (maltodextrin, β-cyclodextrin and their combinations with gum arabic in the ratio 1:1 and 3:1, *w*/*w*) and the sample:carrier ratio (1:1, 1:2, 1:3, *w*/*w*) on the physicochemical properties of the obtained powders (process yield, moisture content, hygroscopicity, solubility, powder morphology) and on the encapsulation and loading capacity, the antioxidant capacity and bioavailability of polyphenols from the powder.

## 2. Materials and Methods

### 2.1. Chemicals

Distilled water was obtained using the Milli-Q water purification system (Millipore, Bedford, MA, USA). Ethanol (96%), sodium acetate (99%) and iron (III) chloride hexahydrate were purchased from Kemika d.d. (Zagreb, Croatia), methanol and sodium bicarbonate from Gram-mol d.o.o. (Zagreb, Croatia), and sodium chloride from Lach-ner (Neratovice, Czech Republic). Maltodextrin (DE 4–7) was procured from Sigma-Aldrich (St. Louis, MO, USA), and β-cyclodextrin, gum arabic, 2,4,6-tri(2-pyridyl)-s-triazine (TPTZ) and 6-hydroxy-2,5,7,8-tetramethylchroman-2-carboxylic acid (Trolox) were from Acros Organics (Geel, Belgium). Pepsin, pancreatin, bile salts and 2,2-diphenyl-1-(2,4,6-trinitrophenyl)hydrazyl (DPPH) were obtained from Sigma-Aldrich (St. Louis, MO, USA) and hydrochloric acid (37%) from Carlo Erba Reagents (Val-de-Reuil, France), while acetic acid was purchased from J.T.Baker (Deventer, The Netherlands). Standards for gallic acid, chlorogenic acid, protocatechuic acid, synaptic acid, ferulic acid, quinic acid, caffeic acid, *p*-coumaric acid, esculetin, quercetin-3-glucoside, kaempferol-3-glucoside, scopoletin and myricetin were obtained from Sigma-Aldrich (St. Louis, MO, USA) and catechin, epigallocatechin gallate, epicatechin gallate, luteolin, naringenin and apigenin were obtained from Extrasynthese (Genay, France).

### 2.2. Material

Commercially available dried nettle leaves (*Urtica dioica* L.) harvested in 2020 (Suban Ltd., Strmec, Croatia) were used for the experiment.

### 2.3. Microwave-Assisted Extraction (MAE)

Prior to extraction, nettle leaves were ground using an electric grinder (Waring WSG30, Sprzęt Laboratoryjny i Medyczny Labpartner KBS, Warszawa, Poland). The extract for encapsulation process was obtained by microwave-assisted extraction in Ethos Easy reactor (Milestone, Sorisole, Italy) which was carried out according to the optimal extraction parameters obtained on nettle leaves (temperature 60 °C, time 5 min, power 300 W) previously determined by Elez Garofulić et al. (2021) [20] with 30% ethanol as solvent, since the powders produced have the potential for application in in value-added and health-promoting functional products. In each extraction cell 10 g of sample, 60 mL of 30% aqueous ethanol solution (*v*/*v*) and magnetic stirrer were added. The cells were placed on the rotor of microwave reactor, the extraction parameters were set and an automatic extraction process was started. Subsequently, extract was filtered through Büchner funnel, collected and stored at −18 °C until spray-dried.

### 2.4. Spray Drying

Spray drying of the nettle extract was carried out using a laboratory device Büchi Mini Spray Dryer B-290 operating in closed mode with an inert loop B295 (Büchi, Switzerland). Nitrogen was used as a drying gas. The dry matter content of the liquid extract was 3.58%. During the process, the following parameters were kept constant: aspirator capacity at 80%, pump capacity at 15% and nozzle cleaner at level 4. Spray drying process was carried out according to the experimental design shown in Table 1. Three different carriers were used to perform the experiment: MD, GA and β-CD, where MD and β-CD were used as single carriers or in combination with GA in the ratio of 1:1 and 3:1 (*w*/*w*), respectively. Also, three different dry matter sample:carrier ratios (1:1, 1:2, 1:3, *w*/*w*) were used. A certain amount of carrier was added to the 100 mL of the water and stirred for 30 min at 50 °C on a magnetic stirrer RT 5 (IKA-Werke, Staufen im Breisgau, Germany) after which a homogeneous solution was mixed with 100 mL of extract. Spray drying was performed at three inlet temperatures: 120, 160 and 200 °C, while corresponding outlet temperatures were around 70, 85 and 100 °C. Powders were produced in duplicate and stored in hermetically sealed plastic containers in desiccator at room temperature until analyzed.

### 2.5. Characterization of the Microcapsules

#### 2.5.1. Process Yield

The yield of the spray drying process was calculated according to the following equation [21]:

(1) $$\mathrm{Process}\;\mathrm{yield}\;\left(\%\right)=\frac{m_{p}}{m_{d}+m_{c}}\;\times \;100$$

where *m_p_* is mass (g) of produced powder, *m_d_* is dry matter (g) of the extract in the volume used for drying and *m_c_* is the mass of carrier (g) added to the extract before the spray drying process.

#### 2.5.2. Moisture Content

The moisture content of encapsulated extracts was determined by drying in an oven Heratherm OMH100 (Thermo Scientific, Dreieich, Germany) at 105 °C to constant weight (AOAC, 1984).

#### 2.5.3. Solubility

The solubility of the encapsulated extracts was determined according to the modified method described by Anderson et al. (1969) [22]. 1 g of encapsulated extract was dissolved in 10 mL of distilled water in a test tube, stirred at vortex mixer for 1 min, thermostated at 37 °C in a B-490 water bath (Büchi, Flawil, Switzerland) for 30 min and then centrifuged (Rotofix 32, Hettich, Kirchlengern, Germany) for 20 min at 5500 rpm. The resulting supernatant was dried in an oven at 105 °C to a constant mass.

Solubility was calculated according to the following equation:

(2) $$\mathrm{Solubility}\;\left(\%\right)=\left(\frac{m_{s}}{m_{p}}\right)\;\times \;100$$

where *m_s_* is the mass (g) of powdered extract obtained by drying the supernatant to constant weight and *m_p_* is the mass (g) of powdered extract taken for analysis.

#### 2.5.4. Hygroscopicity

The hygroscopicity of the encapsulated nettle extracts was determined by the method described by Tonon et al. (2008) [10]. A mass of 1 g of microcapsules was placed in an open Petri dish in a desiccator containing saturated NaCl solution (75.29% humidity) for 7 days at 25 °C. After 7 days, the sample was weighed and hygroscopicity was expressed as grams of adsorbed moisture per 100 g of microcapsules (g 100 g^−^^1^) according to the following equation:

(3) $$\mathrm{Hygroscopicity}\;\left(g/100\;g\right)=\frac{m_{7}\;-\;m_{0}}{m_{0}}\;\times \;100$$

where *m*_7_ is the mass (g) of weighed microcapsules after 7 days and *m*_0_ initial mass (g) of microcapsules.

#### 2.5.5. Encapsulation and Loading Capacity

Encapsulation capacity is determined through the ratio of surface and total phenolic compounds in microcapsules, according to the method of Robert et al. (2010) [23] and loading capacity is determined through amount of total phenolic compounds in microcapsules and weight of microcapsules after spray drying [24].

For extraction of total polyphenols, 0.2 g of powder was mixed with 2 mL of methanol:acetic acid:water solvent (50:8:42, *v*/*v*/*v*) in a test tube. The mixture was stirred on a vortex mixer for 1 min and extracted in an ultrasonic bath at room temperature for 20 min. After extraction, the mixture was centrifuged at 3000 rpm for 10 min. The content of total polyphenols was determined using the Folin–Ciocalteu method reagent [25].

To extract the surface polyphenols 0.2 g of powder was mixed with 2 mL of ethanol:methanol solvent (50:50, *v*/*v*) and stirred on a vortex mixer for 1 min and centrifuged at 3000 rpm for 10 min. The supernatant was filtered through a filter paper and the surface polyphenol content was determined in the same way as total polyphenols.

The encapsulation capacity is calculated through the ratio of surface and total polyphenols according to the following formula:

(4) $$\mathrm{EC}\left(\%\right)=\left(\frac{\mathrm{TP}-\mathrm{SP}}{\mathrm{TP}}\right)\;\times \;100$$

where TP is concentration of total polyphenols (mg gallic acid g^−^^1^) and SP is concentration of surface polyphenols (mg gallic acid g^−^^1^).

The loading capacity is calculated through the ratio of total polyphenols and weight of microcapsules after spray drying according to the following formula:

(5) $$\mathrm{LC}\;\left(\%\right)=\frac{\mathrm{TP}}{\mathrm{MC}}\;\times \;100$$

where TP is amount of polyphenols in microcapsules (g) and MC is weight of microcapsules (g) after spray drying.

#### 2.5.6. Bioavailability

Bioavailability was determined according to in vitro method described by McDougall et al. (2007) [26] and Gunathilake et al. (2018) [27] with some modifications. In the first phase, gastric conditions were simulated by mixing 250 mg of the powder with 10 mL of 0.9% NaCl solution and 800 μL of 40 mg/mL pepsin dissolved in 0.1 M HCl in Falcon tubes. Samples were adjusted to pH 2 with 0.1 M HCl and incubated at 37 °C for one hour with shaking at 100 rpm. Then, 2 mL aliquot was taken from the tube to determine the polyphenol content. The intermediate phase simulated the transition from the stomach to the small intestine, where 1 mL of 0.9% NaCl and 1 mL of 0.5 M NaHCO_3_ are added to the dialysis membranes (6–8 kDa) and returned to the gastric solution. Samples were incubated at 37 °C at 100 rpm for 45 min, and then adjusted to pH 6.5 by addition of 1M NaHCO_3_. In the final phase, conditions in the small intestine were simulated by adding 2.5 mL of pancreatin (2 mg/mL)-bile salt (12 mg/mL) solution to the samples at adjusted pH and incubating the samples for 2 h at 37 °C with shaking at 100 rpm. Subsequntly, 2 mL aliquots were taken from the membrane and tube to determine the polyphenol content by Folin–Ciocalteu method.

#### 2.5.7. Antioxidant Capacity

Antioxidant capacity of nettle encapsulated extracts was determined by two types of assays. Both assays were determined according to the method described by Dobroslavić et al. (2022) [28].

##### FRAP (Ferric Reducing Antioxidant Power) Assay

The FRAP reagent was prepared by mixing 0.3 M sodium acetate buffer, 10 mM TPTZ (2,4,6-tripyridyl-s-triazine) solution dissolved in 40 mM hydrochloride acid and an aqueous solution of 20 mM iron (III) chloride hexahydrate in a 10:1:1 ratio. In addition, 240 μL of distilled water, 80 μL of sample and 2080 μL of FRAP reagent were added to the glass test tubes, stirred in vortex mixer and thermostatted at 37 °C for 5 min. Then, the absorbance was measured at 593 nm using a spectrophotometer. A calibration curve (y = 0.0013) was prepared using Trolox standard solutions (25–1000 µM).

##### DPPH Radical Scavenging Assay

Prior to analysis, a 0.2 mM DPPH (2,2-diphenyl-1-picrylhydrazyl radical) solution in methanol was prepared. Then, 0.75 mL of the sample and 1.5 mL of the 0.2 mM DPPH solution were added to the glass test tubes. The tubes were placed in the dark at room temperature for 20 min after which the absorbance was measured at 517 nm using a spectrophotometer. A calibration curve (y = −0.008x + 1.3476) was prepared using Trolox standard solutions (10–150 µM).

#### 2.5.8. Scanning Electron Microscopy (SEM)

The morphology of the microcapsules was studied using the high-resolution field emission scanning electron microscope (SEM) JSM-7000F (Jeol, Tokyo, Japan) at the Ruđer Bošković Institute, Division of Materials Chemistry, Zagreb, Croatia. Nettle powder samples were deposited in a thin layer on a carbon tape on the sample holder of the electron microscope to fix them and ensure electrical contact with the rest of the instrument. Images were acquired with an accelerating voltage of 5.0 kV at a standard distance of the objective from the sample (WD = 10 mm), and photomicrographs were taken of each sample at 2000× magnification. A secondary electron detector was used to produce the micrograph or image. The morphological characteristics were studied on the microcapsules with the highest polyphenol encapsulation capacity prepared using different carriers at 160 °C and a ratio of dry matter of extract and carriers 1:3.

#### 2.5.9. UPLC-MS/MS Analysis of Polyphenols

Prior to UPLC analysis, 1 g of powder was dissolved in 10 mL of 80% methanol solution and extracted for 20 min at 50 °C in an ultrasonic bath. The obtained extract was filtered through 0.45 μm PTFE membrane filter. Identification and quantification of polyphenols of nettle leaf extract powder with the highest encapsulation capacity was performed by ultra-high performance liquid chromatography with mass spectrometry (UPLC/MS-MS) (Agilent 6430 Triple Quad LC/MS, Agilent Technologies, Santa Clara, CA, USA). Analytes were ionized using an ESI ion source with nitrogen as an inert gas (temperature 300 °C, flow rate 11 L h^−1^), capillary voltage +4 −3.5 kV^−1^ and nebulizer pressure set at 40 psi. The mass spectrometer was connected to a UPLC system (Agilent series 1290 RRLC instrument) which consisted of a binary pump, an autosampler and a column thermostat. Reverse phase separation was performed on Zorbax Eclipse Plus C18 columns 100 × 2.1 mm with a particle size of 1.8 μm (Agilent, Santa Clara, CA, USA). The column temperature was set at 35 °C, and the injection volume was 2.5 μL. Solvent composition and gradient parameters were as previously described by Elez Garofulić et al. (2018) [29]. Software was used for instrument control and data processing Agilent MassHunter Workstation (ver. B.04.01). The identification of phenolic compounds was carried out by comparing the retention time of separated compounds (Rt) with the retention times of standards, polarity and comparing the characteristic values of precursor ions (*m*/*z*) and fragment ions (*m*/*z*) that are specific for each individual compound.

Quantitative determination was carried out using the calibration curves of the standards, where *p*-hydroxybenzoic acid was calculated as gallic acid equivalent and genistic acid according to protocatechuic acid. Quercetin, isorhamnetin, quercetin pentoside, quercetin 3-*O*-rhamnoside, quercetin acetyl-hexoside, quercetin pentosyl-hexoside, quercetin-acetyl-rutinoside, isorhamnetin 3-*O*-rutinoside and quercetin 3-*O*-rutinoside were calculated according to quercetin-3-glucoside, kaempferol 3-*O*-rutinoside, kaempferol pentoside, kaempferol rhamnoside, kaempferol pentosylhexoside and kaempferol according to kaempferol-3-glucoside, epicatehin according to catechin, apigenin 7-*O*-glucoside and genistein according to apigenin, while umbelliferone was expressed as scopoletin equivalent. All analyses have been performed in a duplicate and concentrations of analyzed compounds are expressed as mg 100 g^−^^1^ of dry matter (dm) (N = 4).

### 2.6. Experimental Design and Statistical Analysis

Statistica 12.0 (StatSoft, Inc., Tulsa, OK, USA) was used for experimental design and statistical data processing. The experiments were designed as mixed full factorial design with 2 factors on three and 1 factor on six levels. The influence of temperature (120, 160 and 200 °C), carrier type (MD, MD:GA (1:1), MD:GA (3:1), β-CD, β-CD:GA (1:1) and β-CD:GA (3:1)) and sample:carrier ratio (1:1, 1:2, 1:3) were observed as independent variables, giving in total 54 experimental runs. The dependent variables (process yield, dry matter, solubility, hygroscopicity, encapsulation and loading capacity) were analyzed by analysis of variance (ANOVA). The normality of the residuals was checked by Shapiro-Wilks test and homoscedasticity by the Levene test. A statistically significant difference was considered at the level of *p* ≤ 0.05 (95% confidence interval), and marginal means were compared using Tukey’s HSD test.

## 3. Results and Discussion

**Figure 1:**
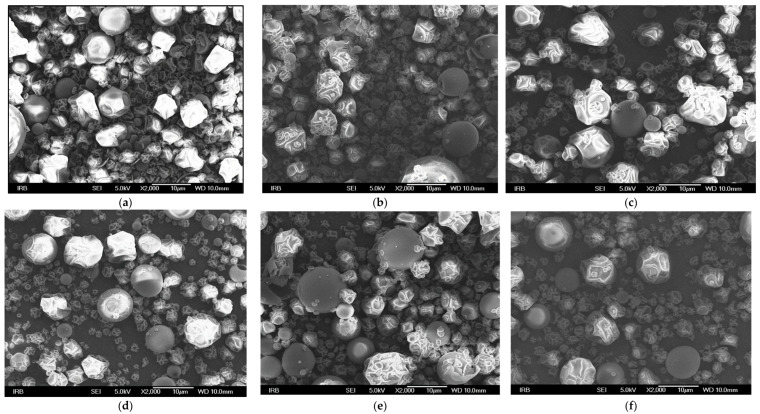
SEM images of the microcapsules of encapsulated nettle leaves extract with different carriers: (**a**) MD (**b**) MD:GA (1:1) (**c**) MD:GA (3:1) (**d**) β-CD (**e**) β-CD:GA (1:1) (**f**) β-CD:GA (3:1) at 160 °C and sample:carrier ratio 1:3.

**Figure 2:**
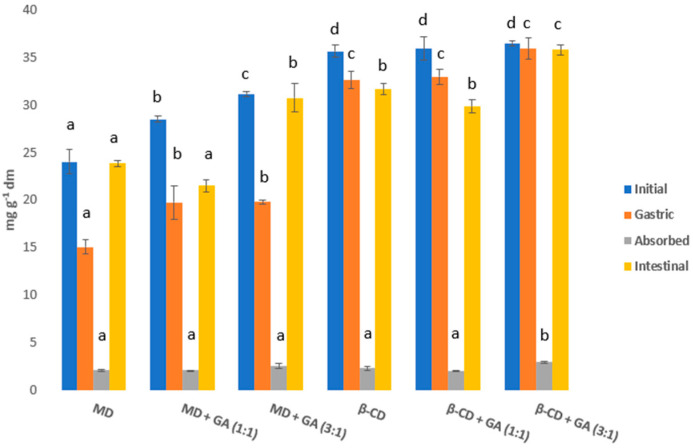
In vitro bioavailability of polyphenols from encapsulated nettle extracts obtained at same conditions (160 °C and sample:carrier ratio 1:3) with different carriers. Values with different letters within phases are statistically different at *p* < 0.05.

**Figure 3:**
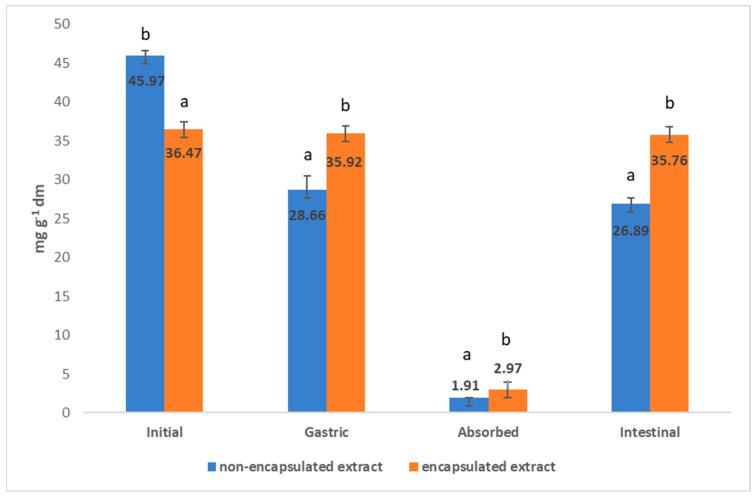
Comparison of the in vitro bioavailability of non-encapsulated extract and encapsulated extract with best properties. Values with different letters within phases are statistically different at *p* < 0.05.

In order to obtain an encapsulated nettle extract with the best physical and chemical properties and with the highest retention and stability of polyphenols, the spray drying encapsulation process needs to be optimized. In addition to the inlet temperature, the type and proportion of the carrier also plays an important role. The experimental design for the production of powders from nettle leaf extract is shown in Table 1 as well as the results of physicochemical properties (process yield, moisture content, solubility, hygroscopicity) and encapsulation and loading capacity of obtained powders. The influence of spray drying parameters on the analyzed properties was tested by ANOVA and is presented in Table 2. Also, the morphology of the selected powders is shown in Figure 1, and antioxidant capacity in Table 3 In addition, the bioavailability of polyphenols in selected powders was studied (Figure 2) and the difference in bioavailability of polyphenols in non-encapsulated and encapsulated extract was demonstrated (Figure 3). Also, UPLC-MS/MS identification and quantification of polyphenols was carried out (Table 4).

### 3.1. Process Yield

As shown in Table 1, the process yield of obtained encapsulated extracts ranged between 64.63–87.23%, with a mean of 75.88%. According to Bhandari et al. (1997) [30], spray drying process can be considered successful when the achieved process yield is above 50%, which was obtained on all encapsulated extracts produced in this study. Losses of powder particles which consequently lead to lower yields, can occur due to particles sticking to the wall of the drying chamber, being pumped off through the outlet air filter, or due to manual operations when collecting powder [31]. The results of spray drying process in this study are relatively high compared to the process yields of other similar plant species [19,32,33,34]. This could be due to the use of different feed compositions, drying conditions, carriers in different proportions and manual operations.

Table 2 shows that both type and proportion of carrier had a statistically significant influence (*p* < 0.01) on process yield, while temperature had no effect (*p* = 0.23). The highest process yield was obtained when MD or its combination with GA (3:1) was used as carrier while the lowest process yield was obtained with β-CD and its combination with GA (3:1). Navarro-Flores et al. (2020) [34] investigated the influence of different carriers on the physicochemical properties of the powder obtained from the methanolic extract of *Crotalaria longirostrata* leaves. The highest process yield in spray drying was obtained when a combination of MD and GA was used as carrier and MD as single carrier. Also, Nadeem et al. (2011) [35] who spray dried the water extract of mountain tea concluded that higher process yield was obtained with MD compared to β-CD and GA. Moreover, the process yield increased when the amount of carrier in the feed solution was increased. This is due to an increase in the total solids in the drying solution, and the addition of a carrier reduces the stickiness so that the particles do not stick to the chamber, which in turn increases the process yield. The same conclusion was reached by Daza et al. (2015) [36] who spray dried Cagaita fruit extracts with GA and inulin, and as the proportion of carriers increased from 10% to 30%, the process yield also increased.

### 3.2. Moisture Content

Moisture content is an important factor affecting the stability of encapsulated extracts. If the moisture content of the powdered extract is relatively low (<5%), this prolongs its shelf life because there is less microbiological contamination, better solubility, and in general greater stability of its properties and thus the possibility of application of the powder for technological purposes [32]. The moisture content of the nettle leaf extract powder was determined in a range of 1.4–7.29% with a mean of 4.02% (Table 1), where most of the powders satisfied the stated thesis. Sablania and Bosco (2018) [37] optimized the spray drying process for *Murraya koenigii* leaves extract and determined moisture content in a range of 3–5.2% which is in accordance with this study. On the other hand, Tran and Nguyen (2018) [38] indicated that a moisture content in lemongrass powders were ranging from 8.49 to 13.11%, which is higher than in this study.

Moisture content was significantly (*p* < 0.01) affected by temperature and carrier content, whereas carrier type did not play a statistically significant role (*p* = 0.28) (Table 2). As the drying temperature increased from 120 to 200°C, the moisture content decreased. Nadeem et al. (2013) [33] stated that the moisture content in sage powders ranged from 3–5%, and decreased with increasing temperature from 145–165 °C, which is consistent with the results of this study. The same conclusion was also reached by the results of other authors [36,39,40]. As the inlet temperature increases, the moisture content decreases, which is due to a faster heat transfer between material to be dried and the heated air. At higher inlet temperatures, there is a greater temperature gradient between the atomized particles and the drying air, resulting in greater driving forces for water evaporation [10,41]. The lowest proportion of moisture content in powder was found at a dry matter ratio of 1:3 of the sample and the carrier. In general, the addition of a carrier decreases the moisture content in the material and thus reduces the proportion of water available for evaporation [42,43].

### 3.3. Solubility

Solubility is also one of the most important parameters of encapsulated extract’ stability. Poor solubility can lead to difficulties in further processing [36]. Consequently, moisture content and particle size affect solubility. Thus, solubility increases with decreasing moisture content and larger particles sink and dissolve faster. As shown in Table 1, the solubility of nettle leaf extract powder ranged from 57.09 to 92.83%, with mean of 89.94%. In the research by Susantikarn and Donlao (2015) [44], the solubility ranged from 94.76–98.53% in green tea powders which is a bit higher than the results of this study.

Moreover, the solubility was significantly (*p* < 0.01) affected by the type of carrier and its proportion, while temperature had no effect (*p* = 0.12). Combinations of the carriers MD and β-CD with GA in the ratio 1:1 proved to be the best combinations and showed the highest solubility of the nettle extract powder, while the lowest solubility was found when β-CD was used as single carrier. Fazaeli et al. (2012) [39] concluded that a mixture of MD and GA is better than using each carrier separately. The increase in solubility by combined carriers is probably due to the chemical structure of the carrier itself. Maltodextrin contains numerous hydroxyl groups that facilitate the dissolution process, while GA has good emulsifying properties and highly branched structure in addition to good solubility [45,46]. The solubility was the lowest when a β-CD was used as a carrier. This is because of β-CD is the least soluble in water of all the carriers observed due to intramolecular hydrogen bonds between the hydroxyl groups of adjacent glucose units. A similar conclusion was reached by Pudziuvelyte et al. (2019) [47] who studied the influence of different carriers on the physicochemical properties of powder from *Elsholtzia ciliate* herb. The highest solubility of the powder was determined with resistant-maltodextrin, and the lowest with β-CD. In addition, increasing the amount of carrier in the drying solution, the solubility also increased. Thus, the highest solubility of the powder was found at a ratio of 1:3 between the dry matter of the extract and the carrier. Daza et al. (2015) [36] investigated the effect of carrier content on the solubility of Cagaita fruit powder. They also concluded that the solubility of the powder increased by increasing the carrier content from 10% to 30%.

### 3.4. Hygroscopicity

Hygroscopicity is a parameter that can be used to predict the behavior of an encapsulated extract during storage and indicates its stability. The results of the research show that the hygroscopicity of the encapsulated nettle leaf extract ranged between 13.35–32.92 g 100 g^−1^ (Table 1) with mean of 21.62 g 100 g^−1^. Zokti et al. (2016) [48] and Susantikarn and Donlao (2015) [44] encapsulated green tea extracts by spray drying and concluded that hygroscopicity values of obtained powders were lower than in this research (3.22–4.71% and 8.61–13.72%).

Carrier type and sample:carrier ratio had a statistically significant effect (*p* < 0.01) on hygroscopicity, while temperature did not (*p* = 0.26). The lowest hygroscopicity was observed in powders when MD and β-CD were used as single carriers, and the value was slightly lower when MD was used as a carrier, while it was higher when they were used in combination with GA. This could be due to the chemical structure of the carriers. Maltodextrin itself has low hygroscopicity and is therefore a very effective carrier for spray drying, and the lower the degree of polymerization of the carrier, the lower the degree of water adsorption, while GA has a branched structure, so water molecules bind more easily to hydroxyl groups in the GA structure [49]. Also, as the proportion of carrier in the solution increased, the hygroscopicity decreased. In general, the addition of a carrier to the material to be dried increases the dry matter content of the material, resulting in the production of a powder that ultimately contains less water and consequently has a lower hygroscopicity [50,51]. Mishra et al. (2013) [52] studied the effect of concentrations of MD on the hygroscopicity of powder obtained from amla juice and Vidović et al. (2014) [32] on *Satureja montana* powder and concluded that the higher the carrier concentration, the lower the hygroscopicity, which is consistent with the results of this study.

### 3.5. Encapsulation and Loading Capacity

The encapsulation capacity, calculated from the ratio of surface and total polyphenols (Table S1), ranged from 95.42–98.67% (Table 1) with mean of 97.20% and the loading capacity ranged between 6.69–20.28%, with mean of 12.01%, showing a very high degree of polyphenol encapsulation under all spray drying conditions applied. Compared to other study conducted on nettle powders [19] it is evident that they got lower values for polyphenol encapsulation capacity (63.23–87.21%), while loading capacity of nettle powder was not recorded in literature data

According to the statistics, the temperature and type of carriers had a statistically significant effect (*p* < 0.01) on the encapsulation capacity, while the proportion of carriers had no effect (*p* = 0.16). On the other side, the type of carrier and their proportion had significant effect (*p* < 0.01) on the loading capacity and temperature did not (*p* = 0.36). The highest encapsulation capacity was observed at the lowest drying temperature of 120 °C and decreased with increasing temperature. Polyphenols are bioactive compounds that are sensitive to external conditions and thus to high temperature where thermal decomposition, polymerization and transformation reactions, can occur. Also, drying at high temperatures directly affects the formation of lower quality products due to color decrease and loss of nutrients [41,53]. Regarding the influence of the carrier on encapsulation capacity, MD and its combinations with GA and β-CD with GA (1:1) proved to be most effective in achieving the best encapsulation capacity, while the lowest capacity was achieved when β-CD was used as single carrier. Navarro-Flores et al. (2020) [34] investigated the microencapsulation efficiency of powders obtained from chipilin leaf extract using different carriers. The lowest encapsulation efficiency of polyphenols was obtained when MD was used as single carrier, while it was significantly higher when combined with other carriers. Watson et al. (2017) [54] concluded that MD and GA exhibit better solubility and are more heat stable than β-CD so these carriers capture the active substance better when the mixture passes through a spray dryer. Moreover, the carrier combination is a better choice than using a single carrier because each carrier contributes to the encapsulation thanks to its structure. In a study by Zokti et al. (2016) [48] on the encapsulation of green tea leaf extract, the combination of MD and GA resulted in a higher encapsulation capacity than the use of a single MD.

### 3.6. SEM Analysis

Figure 1a–f show SEM microstructural analyses of nettle leaf powders with the highest encapsulation capacity prepared under the same drying conditions but with different carriers. The particle size was not uniform, ranging from 2 to 12 μm. In all the images, it can be seen that the some microparticles had a regular round spherical shape and some had depressions on the surface without cracks, which means that the encapsulation was well performed.

Comparing the particle sizes obtained with MD and β-CD, it can be seen that the larger particles were obtained with MD which is in agreement with the research by Chong et al. (2014) [55] who encapsulated betacyanins from *Amaranthus gangeticus* with MD and β-CD. An expansion in the particles size occurs due to the addition of carriers and the inability of water to evaporate rapidly because the carrier retains them [35] and rapid drying at high temperatures leads to the formation of wrinkles and dents on the surface of the microparticles. Kalajahi and Ghandiha (2022) [19] who encapsulated nettle extract using MD as a carrier, concluded that MD resulted in the formation of particles with irregular surface but without cracks and holes. The same conclusion was obtained by Pudziuvelyte et al. (2019) [47] who studied the morphology of *Elsholtzia ciliata* herb powder particles and demonstrated that MD resulted in smoother particles compared to GA and β-CD.

### 3.7. Bioavailability of Polyphenols

Bioavailability is the amount of a nutrient or bioactive ingredient that the human body can store or use in various metabolic processes, and it is necessary to determine it because the beneficial effects of the bioactive ingredient depend on its bioavailability in the body.

To study the bioavailability of polyphenols, the group of powders with the highest encapsulation capacity was selected, prepared under the same spray drying conditions (160 °C and sample:carrier ratio 1:3), using different types of carriers. The concentrations of phenolic compounds in the initial samples of the encapsulated nettle leaf extracts and in all phases of the simulated digestion are shown in Figure 2. The concentrations of polyphenols in the initial powders ranged from 24.01 to 36.47 mg g^−1^ dm of extract. The polyphenol concentrations in the gastric phase were slightly lower (15.09–35.92 mg g^−1^ dm of extract) than in the initial sample in all samples. The results show that in the gastric phase 62.85–98.49% of polyphenols were available for bioavailability, based on the amount in the initial powder, and the values of absorbed polyphenols ranged from 2.03–2.97 mg g^−1^ dm of the extract, and an average 6.16–13.95% were absorbed into the bloodstream through the small intestine. This is consistent with the literature data stating that a very small amount of polyphenols (5–10%) is absorbed during the digestive phase in the small intestine, while most of it is absorbed in the colon due to chemical modification carried out by the microorganisms present there [17]. The largest amount of polyphenols (75.42–99.37%) was released during the intestinal phase and was available for degradation by the microflora in the colon. Shahidi and Peng (2018) [56] concluded that in a three-phase in vitro digestion test, the greatest release of phenolic compounds occurs in the intestinal phase. Zokti et al. (2016) [48] considered that most phenolic compounds are released in the intestinal phase because the interactions between water molecules and amorphous powder microparticles are strong, thereby increasing the solubility of the phenolic compounds. Similarly, dissociation of powder microparticles, which occurs due to the change in pH as the contents pass from the gastric to the intestinal phase, contributes to the release of phenolic compounds. Ydjedd et al. (2017) [57] also demonstrated that the concentration of phenolic compounds of the encapsulated carob extract gradually increased during digestion, with the highest concentrations recorded in the intestinal phase. As can be seen, powders produced with β-CD and its mixture with GA had higher concentration of polyphenols during the bioavailability process than those produced with MD and GA. Grgić et al. (2020) [17] stated that the use of cyclodextrin as a carrier in the encapsulation process increased the solubility of active ingredients and permeability through the intestinal membrane and contributed to higher bioavailability of the encapsulated compound, while GA formed a dry layer and prevented contact between the core and air.

The sample obtained at 160 °C with carrier β-CD:GA (3:1) and sample:carrier ratio 1:3 showed the highest concentrations of polyphenols at all stages of the in vitro digestion test (Figure 2). Also, the mentioned encapsulated extract showed a high retention of polyphenols (79.33%) compared to the initial extract. Tuan et al. (2016) [58] encapsulated guava leaves extracts with mixture of MD and GA. Only 48% of polyphenols was encapsulated by spray drying when compared with polyphenols before encapsulation. On the other hand, Jovanović et al. (2021) [59] concluded that polyphenols retention of willowherb leaves encapsulated extract was 75.80% when 20% MD was used as carrier, which is still lower than in this research. In accordance, in this study the mentioned encapsulated extract was compared with the non-encapsulated extract for the concentrations of phenolic compounds during the in vitro bioavailability phases (Figure 3). As it can be seen, the bioavailability of the phenolic compounds of the encapsulated powder is higher than that of the non-encapsulated extract, indicating that encapsulation effectively protects the phenolic compounds from the adverse conditions in the gastrointestinal tract. For the non-encapsulated extract, it is observed that the polyphenol concentration decreased at each digestive stage due to the influence of enzymes and digestive fluids on polyphenol degradation. On the other hand, the encapsulated extract retained a high level of polyphenolic compounds at each stage, precisely because the carrier protects them from adverse conditions. Comparing the concentration of phenolic compounds of the initial extract and powder with the concentration of the absorbed compounds, a significantly higher absorption of phenolic compounds of the encapsulated powder was observed compared to the non-encapsulated extract. The concentration of phenolic compounds of the initial non-encapsulated extract was 45.97 mg g^−1^ dm of extract, and in an in vitro digestion test, 1.91 mg g^−1^ dm of extract was absorbed, corresponding to 4.16%. On the other hand, the concentration of initial encapsulated extract was 36.47 mg g^−1^ dm of extract and was absorbed by 2.97 mg g^−1^ dm of extract, corresponding to 8.13% and was two-fold higher than non-encapsulated extract. In order to study the full bioavailability of phenolic compounds, it would be necessary to simulate digestion in the colon, since in the work of Grgić et al. (2020) [17] and Bonetti et al. (2016) [60] it was found that the highest concentration of phenolic compounds was absorbed in the colon.

### 3.8. Antioxidant Capacity

The antioxidant capacity of the group of powders with the highest encapsulation capacity prepared under the same spray drying conditions (160 °C and sample: carrier ratio 1:3), and using different carriers was investigated using FRAP and DPPH tests and is shown in Table 4. The FRAP values of the nettle leaf powder were in the range of 6.13–11.04 mmol TE 100g^−1^ dm of the extract and for DPPH ranged from 9.10 to 15.57 mmol TE 100g^−1^ dm of th extract. In both tests, the lowest value was obtained when MD was used as the carrier and the highest value when β-CD:GA in the ratio of 3:1 was used as the carrier. Bhusari and Kumar (2014) [61] spray dried tamarind pulp and investigated how the carrier type affects the antioxidant activity. They concluded that powders with GA had higher antioxidant activity than those obtained with MD. This is due the fact that GA has a little of protein content which could contribute to the increase in antioxidant capacity [62]. Sharayei et al. (2020) [63] encapsulated pomegranate peel extract with MD and β-CD and concluded that the powder obtained with β-CD had higher total polyphenol concentration and antioxidant capacity. Other authors also concluded that cyclodextrins as carriers improved antioxidant capacity of phenolic compounds [64]. This can be attributed to the different structure of studied carriers and the influence of drying parameters [15]. The values of antioxidant capacity follow the values of total phenols, but not completely, which means that the antioxidant capacity is influenced by other components besides phenols, such as chlorophylls and carotenoids [6].

### 3.9. UPLC-MS/MS Identification and Quantification of Polyphenols

For the encapsulated extract with the highest encapsulation efficiency, obtained at 160 °C with β-CD:GA (3:1) and a sample:carrier ratio of 1:3, a complete characterization of the polyphenolic compounds was performed by UPLC-MS/MS (Table 3). A detailed description of identification pathways of phenolic compounds lacking standards from nettle leaves was described by Repajić et al. [6]. A total of 40 compounds belonging to the classes of flavonols, flavan-3-ols, flavones, isoflavones, flavanones, coumarins, benzoic acids, cinnamic acids and other acids were identified (Figure S1). The most abundant group were cinnamic acids (80% of total polyphenols) with cinnamic acid being the major compound (828.47 mg 100 g^−1^), followed by caffeic acid (194.82 mg 100 g^−1^). Other authors also reported that cinnamic acids were predominant group in nettle leaves [6,20,65,66]. The second most dominant group of encapsulated nettle leaves were other phenolic acids (quinic acid), followed by flavan-3-ols, benzoic acids and flavonols and the least respresented group were flavanones. Flavonols were the most numerous groups with 16 phenolic compounds, where kaempferol was the most abundant compound (9.44 mg 100 g^−1^). Elez Garofulić et al. (2021) [20] also concluded that kaempferol was the most dominant polyphenol in flavonol group in nettle leaves. Comparing the identification and quantification of phenolic compounds of nettle leaves with other studies, differences may occur due to different growing conditions and harvesting times [6], different preparation of the extract [20], and in this case, due to the effects of encapsulation on the polyphenols.

## 4. Conclusions

The results of this study emphasized the necessity of careful selection of encapsulation parameters in order to obtain encapsulated nettle leaf extract with desirable physicochemical properties and preserved polyphenolic content. Therefore, the highest process yield was obtained at 160 °C, when MD was used as carrier, in sample:carrier ratio 1:3. The lowest moisture was achieved at 200 °C with β-CD and sample:carrier ratio 1:2, the highest solubility at 120 °C with MD and sample:carrier ratio 1:2, while the lowest hygroscopicity was achieved at 200 °C with MD:GA (3:1) and sample:carrier ratio 1:3. For the encapsulation and loading capacity, 160 °C, β-CD:GA (3:1) and sample:carrier ratio 1:3 were conditions for obtaining highest values. Since the positive effects of polyphenols significantly depend on their bioavailability in the human organism, it has been proven that encapsulation of nettle leaf extracts enabled a two-fold increase in polyphenol bioavailability. The present study showed that nettle leaf extract powders are a rich source of polyphenols and have high antioxidant capacity. In UPLC-MS /MS profiling, 40 phenolic compounds were identified, with cinnamic acids being the most abundant. Therefore, spray drying encapsulation of nettle leaf extract showed to be a promising tool for preservation and stabilization of valuable antioxidants with increased bioavailability, thus enabling their application in functional food products.

## Figures and Tables

**Table 1:** Physicochemical properties and encapsulation capacity of nettle leaves extract powders obtained with different carrier agents added in different ratios under different temperatures.

Sample	Carrier	Sample: Carrier Ratio	Temperature (°C)	Process Yield (%)	Moisture Content (%)	Solubility (%)	Hygroscopicity (g 100 g^−1^)	Encapsulation Capacity (%)	Loading Capacity (%)
**1**	**MD**	**1:1**	**120**	74.92 ± 0.29	3.8 ± 0.62	82.21 ± 0.46	23.03 ± 0.11	97.61 ± 0.04	15.32 ± 0.09
**2**	**MD:GA (1:1)**			73.52 ± 0.03	7.29 ± 0.67	82.01 ± 0.79	25.93 ± 0.49	97.07 ± 0.16	14.29 ± 0.32
**3**	**MD:GA (3:1)**			73.12 ± 0.35	5.18 ± 0.49	87.46 ± 0.14	29.96 ± 0.25	97.37 ± 0.09	16.27 ± 0.16
**4**	**β-CD**			72.96 ± 0.82	4.55 ± 0.75	76.91 ± 1.34	24.26 ± 0.10	97.37 ± 0.26	17.33 ± 1.30
**5**	**β-CD:GA (1:1)**			70.87 ± 0.36	5.8 ± 0.37	90.63 ± 0.60	30.21 ± 1.21	97.59 ± 0.04	16.35 ± 0.22
**6**	**β-CD:GA (3:1)**			70.35 ± 0.99	5.49 ± 0.85	75.18 ± 0.72	27.66 ± 0.00	97.64 ± 0.09	17.67 ± 0.15
**7**	**MD**	**1:2**		75.22 ± 0.68	5.36 ± 0.01	83.75 ± 0.54	13.35 ± 0.65	98.51 ± 0.04	9.81 ± 0.25
**8**	**MD:GA (1:1)**			73.83 ± 1.23	6.88 ± 0.35	85.18 ± 0.51	15.04 ± 0.27	98.66 ± 0.06	9.32 ± 0.12
**9**	**MD:GA (3:1)**			77.61 ± 0.09	3.27 ± 0.64	80.95 ± 0.12	21.77 ± 0.42	97.76 ± 0.04	10.59 ± 0.04
**10**	**β-CD**			74.76 ± 0.45	4.87 ± 0.21	88.86 ± 0.24	14.11 ± 0.32	97.46 ± 0.23	10.56 ± 0.14
**11**	**β-CD:GA (1:1)**			75.93 ± 0.42	4.12 ± 0.28	88.35 ± 0.68	19.49 ± 0.74	97.76 ± 0.11	11.37 ± 0.07
**12**	**β-CD:GA (3:1)**			73.49 ± 0.13	4.81 ± 0.53	78.98 ± 0.29	20.29 ± 0.52	97.51 ± 0.11	10.98 ± 0.48
**13**	**MD**	**1:3**		77.17 ± 0.53	4.11 ± 0.46	90.41 ± 0.24	14.57 ± 0.75	98.40 ± 0.04	6.69 ± 0.07
**14**	**MD:GA (1:1)**			76.25 ± 0.59	3.74 ± 0.06	89.27 ± 0.57	18.08 ± 0.21	98.65 ± 0.09	7.24 ± 0.04
**15**	**MD:GA (3:1)**			82.42 ± 0.86	4.9 ± 0.6	91.32 ± 0.06	22.71 ± 0.84	97.72 ± 0.20	7.55 ± 0.08
**16**	**β-CD**			74.90 ± 0.44	4.49 ± 0.89	86.10 ± 0.75	14.60 ± 0.65	95.42 ± 0.13	11.02 ± 0.09
**17**	**β-CD:GA (1:1)**			81.47 ± 0.76	3.97 ± 0.52	91.43 ± 0.44	19.27 ± 0.54	97.84 ± 0.03	9.47 ± 0.04
**18**	**β-CD:GA (3:1)**			77.00 ± 0.45	3.48 ± 0.75	89.25 ± 0.79	18.19 ± 0.64	96.82 ± 0.05	9.60 ± 0.08
**19**	**MD**	**1:1**	**160**	80.35 ± 0.61	3.55 ± 0.83	85.01 ± 0.75	27.59 ± 0.36	98.14 ± 0.04	19.07 ± 0.26
**20**	**MD:GA (1:1)**			73.61 ± 0.10	4.9 ± 0.33	90.19 ± 0.03	32.92 ± 0.47	97.03 ± 0.07	14.31 ± 0.25
**21**	**MD:GA (3:1)**			75.06 ± 0.66	4.88 ± 0.64	87.28 ± 0.84	32.11 ± 0.62	97.26 ± 0.07	17.10 ± 0.06
**22**	**β-CD**			68.98 ± 0.42	4.9 ± 0.89	57.09 ± 0.15	25.55 ± 0.62	96.61 ± 0.05	12.88 ± 0.08
**23**	**β-CD:GA (1:1)**			64.63 ± 0.71	6.92 ± 0.6	88.24 ± 0.78	31.41 ± 0.23	97.11 ± 0.02	15.29 ± 0.19
**24**	**β-CD:GA (3:1)**			70.38 ± 0.23	4 ± 0.62	78.20 ± 0.92	29.36 ± 0.84	96.55 ± 0.03	14.50 ± 0.20
**25**	**MD**	**1:2**		77.56 ± 0.40	2.31 ± 0.49	89.59 ± 0.09	19.98 ± 0.41	97.51 ± 0.03	9.30 ± 0.04
**26**	**MD:GA (1:1)**			75.92 ± 0.12	2.66 ± 0.64	87.88 ± 0.86	24.21 ± 0.91	97.68 ± 0.09	9.93 ± 0.13
**27**	**MD:GA (3:1)**			80.16 ± 0.12	6.43 ± 0.3	87.63 ± 0.17	21.98 ± 0.40	97.31 ± 0.04	10.07 ± 0.07
**28**	**β-CD**			74.41 ± 0.08	3.52 ± 0.84	62.13 ± 0.96	15.88 ± 0.35	96.92 ± 0.13	11.56 ± 0.05
**29**	**β-CD:GA (1:1)**			78.52 ± 0.84	2.48 ± 0.77	88.64 ± 0.22	21.85 ± 0.28	97.72 ± 0.05	12.61 ± 0.02
**30**	**β-CD:GA (3:1)**			73.85 ± 0.32	4.45 ± 0.11	80.82 ± 0.41	21.79 ± 0.87	97.23 ± 0.02	12.96 ± 0.03
**31**	**MD**	**1:3**		87.23 ± 0.41	2.98 ± 0.76	87.77 ± 0.85	13.93 ± 0.13	97.91 ± 0.09	8.69 ± 0.20
**32**	**MD:GA (1:1)**			84.54 ± 0.93	3.61 ± 0.09	91.11 ± 0.80	15.92 ± 0.49	98.16 ± 0.15	7.61 ± 0.08
**33**	**MD:GA (3:1)**			78.59 ± 0.86	4.08 ± 0.55	83.63 ± 0.72	20.13 ± 0.65	97.90 ± 0.12	9.66 ± 0.18
**34**	**β-CD**			76.01 ± 0.80	2.65 ± 0.71	86.53 ± 0.04	16.37 ± 0.43	95.68 ± 0.07	13.20 ± 0.02
**35**	**β-CD:GA (1:1)**			81.86 ± 0.71	3.79 ± 0.66	89.51 ± 0.47	22.57 ± 0.37	97.47 ± 0.11	10.42 ± 0.08
**36**	**β-CD:GA (3:1)**			80.28 ± 0.70	3.6 ± 0.1	90.69 ± 0.73	18.25 ± 0.44	98.67 ± 0.07	20.28 ± 0.17
**37**	**MD**	**1:1**	**200**	76.32 ± 1.05	4.05 ± 0.61	90.24 ± 0.62	20.82 ± 0.56	96.68 ± 0.10	13.11 ± 0.05
**38**	**MD:GA (1:1)**			74.51 ± 0.80	4.82 ± 0.45	91.37 ± 0.14	22.75 ± 0.55	95.52 ± 0.20	11.68 ± 0.07
**39**	**MD:GA (3:1)**			72.41 ± 0.04	3.87 ± 0.19	88.96 ± 0.55	28.11 ± 0.47	96.56 ± 0.00	14.59 ± 0.05
**40**	**β-CD**			69.50 ± 0.55	3.27 ± 0.03	60.24 ± 0.53	24.23 ± 0.72	96.81 ± 0.14	16.10 ± 0.33
**41**	**β-CD:GA (1:1)**			72.57 ± 0.18	2.69 ± 0.35	90.36 ± 0.17	29.76 ± 0.85	96.96 ± 0.14	17.13 ± 0.53
**42**	**β-CD:GA (3:1)**			68.96 ± 1.03	1.64 ± 0.55	75.80 ± 1.01	26.01 ± 0.61	96.54 ± 0.18	15.41 ± 0.37
**43**	**MD**	**1:2**		78.25 ± 0.68	3.92 ± 0.6	86.89 ± 0.06	15.77 ± 0.67	96.68 ± 0.00	9.20 ± 0.12
**44**	**MD:GA (1:1)**			76.60 ± 0.00	3.43 ± 0.27	89.37 ± 0.73	17.47 ± 0.53	97.32 ± 0.03	11.20 ± 0.10
**45**	**MD:GA (3:1)**			79.42 ± 0.71	4.13 ± 0.4	90.72 ± 0.59	21.31 ± 0.61	97.48 ± 0.03	10.19 ± 0.03
**46**	**β-CD**			67.96 ± 1.41	1.4 ± 0.04	64.80 ± 0.77	19.08 ± 0.16	96.03 ± 0.08	11.31 ± 0.08
**47**	**β-CD:GA (1:1)**			76.18 ± 0.94	3.87 ± 0.53	90.48 ± 0.85	26.40 ± 0.26	96.31 ± 0.06	11.49 ± 0.08
**48**	**β-CD:GA (3:1)**			74.78 ± 0.41	3.5 ± 0.76	81.52 ± 0.92	24.01 ± 1.18	96.00 ± 0.01	11.20 ± 0.07
**49**	**MD**	**1:3**		83.42 ± 0.07	3.11 ± 0.3	83.07 ± 0.30	13.64 ± 0.55	96.13 ± 0.03	7.16 ± 0.09
**50**	**MD:GA (1:1)**			83.29 ± 0.48	3.38 ± 0.11	91.52 ± 0.75	17.10 ± 0.25	97.47 ± 0.09	7.75 ± 0.03
**51**	**MD:GA (3:1)**			81.62 ± 0.56	2.63 ± 0.72	92.83 ± 0.67	19.90 ± 1.30	97.45 ± 0.16	7.95 ± 0.03
**52**	**β-CD**			78.54 ± 0.81	2.66 ± 0.22	87.24 ± 0.84	15.63 ± 0.38	95.57 ± 0.10	13.70 ± 0.02
**53**	**β-CD:GA (1:1)**			75.44 ± 0.31	4.32 ± 0.15	90.05 ± 1.15	23.17 ± 0.90	97.37 ± 0.03	9.14 ± 0.24
**54**	**β-CD:GA (3:1)**			70.07 ± 0.28	2.51 ± 0.78	91.22 ± 0.92	18.01 ± 0.45	96.16 ± 0.09	9.24 ± 0.01
	**MEAN**			75.88	4.02	89.94	21.62	97.20	12.01

Results are expressed as mean ± SD (N = 4).

**Table 2:** Influence of spray drying parameters on the process yield, moisture content, solubility, hygroscopicity, encapsulation and loading capacity of nettle leaves extract powders.

	N	Process Yield (%)	Moisture Content (%)	Solubility (%)	Hygroscopicity (g 100 g^−1^)	Encapsulation Capacity (%)	Loading Capacity (%)
**Temperature (°C)**		*p* = 0.23	*p* < 0.01	*p* = 0.12	*p* = 0.26	*p* < 0.01	*p* = 0.36
**120**	36	75.32 ± 0.51 ^a^	4.78 ± 0.19 ^c^	85.46 ± 0.84 ^a^	20.70 ± 0.89 ^a^	97.62 ± 0.13 ^c^	11.75 ± 0.58 ^a^
**160**	36	76.77 ± 0.91 ^a^	3.98 ± 0.22 ^b^	84.00 ± 1.57 ^a^	22.88 ± 0.97 ^a^	97.38 ± 0.11 ^b^	12.74 ± 0.58 ^a^
**200**	36	75.55 ± 0.78 ^a^	3.29 ± 0.15 ^a^	85.37 ± 1.54 ^a^	21.29 ± 0.76 ^a^	96.61 ± 0.11 ^a^	11.53 ± 0.49 ^a^
**Carrier**		*p* < 0.01	*p* = 0.28	*p* < 0.01	*p* < 0.01	*p* < 0.01	*p* < 0.01
**MD**	18	78.94 ± 0.94 ^b^	3.69 ± 0.21 ^a^	86.55 ± 0.73 ^ab^	18.08 ± 1.16 ^a^	97.51 ± 0.19 ^b^	10.93 ± 0.93 ^ab^
**MD:GA (1:1)**	18	76.90 ± 0.96 ^ab^	4.52 ± 0.37 ^a^	88.66 ± 0.74 ^c^	21.05 ± 1.35 ^ab^	97.51 ± 0.23 ^b^	10.37 ± 0.62 ^a^
**MD:GA (3:1)**	18	77.82 ± 0.83 ^b^	4.38 ± 0.27 ^a^	87.87 ± 0.86 ^b^	24.22 ± 1.05 ^b^	97.41 ± 0.09 ^b^	11.55 ± 0.80 ^ab^
**β-CD**	18	73.11 ± 0.83 ^a^	3.59 ± 0.30 ^a^	74.43 ± 3.04 ^a^	18.86 ± 1.05 ^a^	96.43 ± 0.21 ^a^	13.07 ± 0.53 ^ab^
**β-CD:GA (1:1)**	18	75.27 ± 1.24 ^ab^	4.22 ± 0.33 ^a^	89.74 ± 0.28 ^c^	24.90 ± 1.07 ^b^	97.35 ± 0.11 ^b^	12.08 ± 0.68 ^ab^
**β-CD:GA (3:1)**	18	73.24 ± 0.86 ^a^	3.72 ± 0.29 ^a^	82.41 ± 1.45 ^ab^	22.62 ± 1.00 ^ab^	97.01 ± 0.13 _ab_	13.54 ± 0.86 ^b^
**Ratio sample:carrier**		*p* < 0.01	*p* < 0.01	*p* < 0.01	*p* < 0.01	*p* = 0.16	*p* < 0.01
**1:1**	36	72.39 ± 0.57 ^a^	4.53 ± 0.13 ^b^	82.08 ± 1.66 ^a^	27.32 ± 0.57 ^b^	97.02 ± 0.10 ^a^	15.47 ± 0.31 ^c^
**1:2**	36	75.80 ± 0.46 ^b^	3.97 ± 0.24 ^ab^	83.70 ± 1.35 ^a^	19.65 ± 0.61 ^a^	97.32 ± 0.12 ^a^	10.76 ± 0.18 ^b^
**1:3**	36	79.45 ± 0.70 ^c^	3.56 ± 0.24 ^a^	89.05 ± 0.46 ^b^	17.89 ± 0.50 ^a^	97.27 ± 0.17 ^a^	9.80 ± 0.54 ^a^

Results are expressed as mean ± SD (N = 4). Values with different letters within column are statistically different at *p* < 0.05.

**Table 3:** Antioxidant capacity of nettle leaf powder obtained at same conditions (160 °C and sample:carrier ratio 1:3) with different carriers.

Sample	Carrier	Sample: Carrier Ratio	Temperature (°C)	FRAP (mmol TE 100g^−1^ dm)	DPPH (mmol TE 100g^−1^ dm)
**31**	**MD**	**1:3**	**160**	6.13 ± 0.18 ^a^	9.10 ± 0.08 ^a^
**32**	**MD:GA (1:1)**			8.57 ± 0.40 ^b^	12.42 ± 0.16 ^b^
**33**	**MD:GA (3:1)**			8.30 ± 0.62 ^b^	12.11 ± 0.03 ^b^
**34**	**β-CD**			9.59 ± 0.16 ^bc^	12.74 ± 0.12 ^b^
**35**	**β-CD:GA (1:1)**			10.55 ± 0.20 ^c^	12.13 ± 0.34 ^b^
**36**	**β-CD:GA (3:1)**			11.04 ± 0.27 ^cd^	15.57 ± 0.01 ^c^

Results are expressed as mean ± SD (N = 4). Values with different letters within column are statistically different at *p* < 0.05.

**Table 4:** Polyphenolic profile including mass spectrometric data and concentration of identified individual compounds of nettle leaves extract powder with the highest encapsulation capacity.

	Mass Spectrometric Data			Concentration (mg 100 g^−1^)
Compound	Rt (min)	Precursor Ion (*m*/*z*)	Fragment Ion (*m*/*z*)	Encapsulated Sample 36
FLAVONOLS				
**Quercetin-acetyl-rutinoside**	11.317	653	303	1.45 ± 0.08
**Isorhamnetin 3-*O*-rutinoside**	1.384	625	317	0.36 ± 0.05
**Quercetin 3-*O*-rutinoside**	11.16	611	303	1.00 ± 0.05
**Quercetin-pentosyl-hexoside**	11.498	597	303	0.11 ± 0.03
**Kaempferol 3-*O*-rutinoside**	2.173	595	287	0.29 ± 0.03
**Kaempferol-pentosyl-hexoside**	11.344	581	287	0.28 ± 0.02
**Quercetin-acetyl-hexoside**	11.511	507	303	0.21 ± 0.04
Kaempferol 3-*O*-glucoside *	2.193	449	287	0.09 ± 0.03
**Quercetin 3-*O*-rhamnoside**	12.013	449	303	0.65 ± 0.10
**Quercetin-pentoside**	9.236	435	303	0.26 ± 0.07
**Kaempferol-rhamnoside**	10.551	433	287	0.63 ± 0.04
**Kaempferol-pentoside**	8.054	419	287	0.12 ± 0.07
**Quercetin**	7.732	301	151	0.01 ± 0.00
**Isorhamentin**	6.265	315	300	0.01 ± 0.00
Myricetin *	1.201	319	273	5.79 ± 0.12
**Kaempferol**	11.58	285	285	9.44 ± 0.04
FLAVAN-3-OLS				
Epigallocatechin gallate *	9.711	459	289, 139	0.31 ± 0.05
Epicatechin gallate *	10.872	443	291	0.09 ± 0.03
**Epicatechin**	12.067	291	139	57.66 ± 0.04
Catechin *	11.127	291	165	0.29 ± 0.07
FLAVONES				
**Apigenin 7-*O*-glucoside**	1.863	433	271	2.00 ± 0.11
Apigenin *	7.025	271	153	3.25 ± 0.05
Luteolin *	1.266	287	153	0.83 ± 0.07
ISOFLAVONES				
**Genistein**	7.65	269	133	2.76 ± 0.11
FLAVANONES				
Naringenin *	1.091	271	151	0.11 ± 0.05
COUMARINS				
**Umbelliferone**	0.803	161	133	0.99 ± 0.09
Esculetin *	1.417	177	133	13.59 ± 0.11
Scopoletin *	0.947	191	176	0.80 ± 0.05
BENZOIC ACIDS				
Protocatechuic acid *	0.807	153	109	14.41 ± 0.43
Gallic acid *	11.292	169	125	2.86 ± 0.12
Syringic acid *	10.037	197	182	0.25 ± 0.04
**Gentisic acid**	1.148	153	109	14.78 ± 0.63
***p*** **-hydroxybenzoic acid**	11.313	137	93	6.70 ± 0.21
CINNAMIC ACIDS				
Chlorogenic acid *	0.909	353	191	1.73 ± 0.05
Sinapic acid *	4.213	223	193	0.25 ± 0.04
Ferulic acid *	6.544	193	178	8.82 ± 0.15
Caffeic acid *	1.414	179	135	194.82 ± 2.92
***p*** -coumaric acid *	3.624	163	119	2.30 ± 0.10
Cinnamic acid *	4.465	147	103	828.47 ± 1.09
OTHER ACIDS				
Quinic acid *	0.786	191	85	109.61 ± 0.24
**TOTAL POLYPHENOLS**				1288.39

* Identification confirmed using authentic standards. Results are expressed as mean ± standard deviation.

## Data Availability

The data presented in this study are available on request from the corresponding author.

## Supplementary Materials

The following supporting information can be downloaded at: https://www.mdpi.com/article/10.3390/foods11182852/s1, Table S1: Concentrations of total and surface polyphenols in nettle leaves extract powders; Figure S1: UPLC-MS/MS chromatogram of nettle leaves extract powder.

## References

[1] Veiga M., Costa E.M., Silva S., Pintado M. (2020). Impact of Plant Extracts upon Human Health: A Review. Crit. Rev. Food Sci. Nutr..

[2] Bornkessel S., Bröring S., Omta S.W.F., van Trijp H. (2014). What Determines Ingredient Awareness of Consumers? A Study on Ten Functional Food Ingredients. Food Qual. Prefer..

[3] Grauso L., Emrick S., Bonanomi G., Lanzotti V. (2019). Metabolomics of the Alimurgic Plants Taraxacum Officinale, Papaver Rhoeas and *Urtica dioica* by Combined NMR and GC–MS Analysis. Phytochem. Anal..

[4] Di Virgilio N., Papazoglou E.G., Jankauskiene Z., di Lonardo S., Praczyk M., Wielgusz K. (2015). The Potential of Stinging Nettle (*Urtica dioica* L.) as a Crop with Multiple Uses. Ind. Crops Prod..

[5] Kukrić Z.Z., Topalić-Trivunović L.N., Kukavica B.M., Matoš S.B., Pavičić S.S., Boroja M.M., Savić A.V. (2012). Characterization of Antioxidant and Antimicrobial Activities of Nettle Leaves (*Urtica dioica* L.). Acta Per. Technol..

[6] Repajić M., Cegledi E., Zorić Z., Pedisić S., Garofulić I.E., Radman S., Palčić I., Dragović-Uzelac V. (2021). Bioactive Compounds in Wild Nettle (*Urtica dioica* L.) Leaves and Stalks: Polyphenols and Pigments upon Seasonal and Habitat Variations. Foods.

[7] Otles S., Yalcin B. (2012). Phenolic Compounds Analysis of Root, Stalk, and Leaves of Nettle. Sci. World J..

[8] Upton R. (2013). Stinging Nettles Leaf (*Urtica dioica* L.): Extraordinary Vegetable Medicine. J. Herb. Med..

[9] Dai J., Mumper R.J. (2010). Plant Phenolics: Extraction, Analysis and Their Antioxidant and Anticancer Properties. Molecules.

[10] Tonon R.V., Brabet C., Hubinger M.D. (2008). Influence of Process Conditions on the Physicochemical Properties of Açai (*Euterpe oleraceae* Mart.) Powder Produced by Spray Drying. J. Food Eng..

[11] Buljeta I., Pichler A., Šimunović J., Kopjar M. (2022). Polysaccharides as Carriers of Polyphenols: Comparison of Freeze-Drying and Spray-Drying as Encapsulation Techniques. Molecules.

[12] Igual M., García-Herrera P., Cámara R.M., Martínez-Monzó J., García-Segovia P., Cámara M. (2022). Bioactive Compounds in Rosehip (Rosa Canina) Powder with Encapsulating Agents. Molecules.

[13] Ferrari C.C., Germer S.P.M., de Aguirre J.M. (2012). Effects of Spray-Drying Conditions on the Physicochemical Properties of Blackberry Powder. Dry Technol..

[14] Murugesan R., Orsat V. (2012). Spray Drying for the Production of Nutraceutical Ingredients-A Review. Food Bioproc. Technol..

[15] Kurozawa L.E., Morassi A.G., Vanzo A.A., Park K.J., Hubinger M.D. (2009). Influence of Spray Drying Conditions on Physicochemical Properties of Chicken Meat Powder. Dry Technol..

[16] Peng Z., Li J., Guan Y., Zhao G. (2013). Effect of Carriers on Physicochemical Properties, Antioxidant Activities and Biological Components of Spray-Dried Purple Sweet Potato Flours. Food Sci. Technol..

[17] Grgić J., Šelo G., Planinić M., Tišma M., Bucić-Kojić A. (2020). Role of the Encapsulation in Bioavailability of Phenolic Compounds. Antioxidants.

[18] Vulić J., Šeregelj V., Kalušević A., Lević S., Nedović V., Šaponjac V.T., Čanadanović-Brunet J., Ćetković G. (2019). Bioavailability and Bioactivity of Encapsulated Phenolics and Carotenoids Isolated from Red Pepper Waste. Molecules.

[19] Kalajahi M.S.E., Ghandiha S. (2022). Optimization of Spray Drying Parameters for Encapsulation of Nettle (*Urtica dioica* L.) Extract. LWT.

[20] Elez Garofulić I., Malin V., Repajić M., Zorić Z., Pedisić S., Sterniša M., Možina S.S., Dragović-Uzelac V. (2021). Phenolic Profile, Antioxidant Capacity and Antimicrobial Activity of Nettle Leaves Extracts Obtained by Advanced Extraction Techniques. Molecules.

[21] Zhang L., Zeng X., Fu N., Tang X., Sun Y., Lin L. (2018). Maltodextrin: A Consummate Carrier for Spray-Drying of Xylooligosaccharides. Food Res. Inter..

[22] Anderson R.A., Anderson R.A., Conway H.F., Pfiefer V.F., Griffin E.L. (1969). Roll and Extrusion-Cooking of Grain Sorghum Grits. Cereal Sci. Today.

[23] Robert P., Gorena T., Romero N., Sepulveda E., Chavez J., Saenz C. (2010). Encapsulation of Polyphenols and Anthocyanins from Pomegranate (*Punica granatum*) by Spray Drying. Int. J. Food Sci. Technol..

[24] Hu L., Zhang J., Hu Q., Gao N., Wang S., Sun Y., Yang X. (2016). Microencapsulation of Brucea Javanica Oil: Characterization, Stability and Optimization of Spray Drying Conditions. J. Drug Deliv. Sci. Technol..

[25] Saénz C., Tapia S., Chávez J., Robert P. (2009). Microencapsulation by Spray Drying of Bioactive Compounds from Cactus Pear (*Opuntia ficus-indica*). Food Chem..

[26] Mcdougall G., Dobson P., Shpiro F., Smith P., Stewart D., Fyffe S. (2005). Assessing Bioavailability of Soft Fruit Polyphenols In Vitro. Int. Symp. Hum. Health Eff. Fruits Veg..

[27] Gunathilake K.D.P.P., Ranaweera K.K.D.S., Rupasinghe H.P.V. (2018). Change of Phenolics, Carotenoids, and Antioxidant Capacity Following Simulated Gastrointestinal Digestion and Dialysis of Selected Edible Green Leaves. Food Chem..

[28] Dobroslavić E., Elez Garofulić I., Šeparović J., Zorić Z., Pedisić S., Dragović-Uzelac V. (2022). Pressurized Liquid Extraction as a Novel Technique for the Isolation of *Laurus nobilis* L. Leaf Polyphenols. Molecules.

[29] Elez Garofulić I., Zorić Z., Pedisić S., Brnčić M., Dragović-Uzelac V. (2018). UPLC-MS2 Profiling of Blackthorn Flower Polyphenols Isolated by Ultrasound-Assisted Extraction. J. Food Sci..

[30] Bhandari B.R., Datta N., Howes T. (1997). Problems Associated with Spray Drying of Sugar-Rich Foods. Dry Technol..

[31] Fang Z., Bhandari B. (2011). Effect of Spray Drying and Storage on the Stability of Bayberry Polyphenols. Food Chem..

[32] Vidović S.S., Vladić J.Z., Vaštag Ž.G., Zeković Z.P., Popović L.M. (2014). Maltodextrin as a Carrier of Health Benefit Compounds in Satureja Montana Dry Powder Extract Obtained by Spray Drying Technique. Powder Technol..

[33] Şahin-Nadeem H., Dinçer C., Torun M., Topuz A., Özdemir F. (2013). Influence of Inlet Air Temperature and Carrier Material on the Production of Instant Soluble Sage (*Salvia fruticosa* Miller) by Spray Drying. Food Sci. Technol..

[34] Navarro-Flores M.J., Ventura-Canseco L.M.C., Meza-Gordillo R., Ayora-Talavera T.D.R., Abud-Archila M. (2020). Spray Drying Encapsulation of a Native Plant Extract Rich in Phenolic Compounds with Combinations of Maltodextrin and Non-Conventional Wall Materials. J. Food Sci. Technol..

[35] Şahin-Nadeem H., Torun M., Özdemir F. (2011). Spray Drying of the Mountain Tea (*Sideritis stricta*) Water Extract by Using Different Hydrocolloid Carriers. Food Sci. Technol..

[36] Daza L.D., Fujita A., Fávaro-Trindade C.S., Rodrigues-Ract J.N., Granato D., Genovese M.I. (2016). Effect of Spray Drying Conditions on the Physical Properties of Cagaita (*Eugenia dysenterica* DC.) Fruit Extracts. Food Bioprod. Process..

[37] Sablania V., Bosco S.J.D. (2018). Optimization of Spray Drying Parameters for *Murraya koenigii* (Linn) Leaves Extract Using Response Surface Methodology. Powder Technol..

[38] Tran T.T.A., Nguyen H.V.H. (2018). Effects of Spray-Drying Temperatures and Carriers on Physical and Antioxidant Properties of Lemongrass Leaf Extract Powder. Beverages.

[39] Fazaeli M., Emam-Djomeh Z., Kalbasi Ashtari A., Omid M. (2012). Effect of Spray Drying Conditions and Feed Composition on the Physical Properties of Black Mulberry Juice Powder. Food Bioprod. Process..

[40] Bastías-Montes J.M., Choque-Chávez M.C., Alarcón-Enos J., Quevedo-León R., Muñoz-Fariña O., Vidal-San-martín C. (2019). Effect of Spray Drying at 150, 160, and 170 °C on the Physical and Chemical Properties of Maqui Extract (Aristotelia Chilensis (Molina) Stuntz). Chil. J. Agric. Res..

[41] Quek S.Y., Chok N.K., Swedlund P. (2007). The Physicochemical Properties of Spray-Dried Watermelon Powders. Chem. Eng. Process..

[42] Gunjal S.D., Shirolkar S.V. (2020). An Overview of Process Parameters and Spray Drying Agents Involved in Spray Drying of Herbal Extracts. Paid. J..

[43] Giovagnoli-Vicuña C., Briones-Labarca V., Romero M.S., Giordano A., Pizarro S. (2022). Effect of Extraction Methods and In Vitro Bio-Accessibility of Microencapsulated Lemon Extract. Molecules.

[44] Susantikarn P., Donlao N. (2016). Optimization of Green Tea Extracts Spray Drying as Affected by Temperature and Maltodextrin Content. Int. Food Res. J..

[45] Phisut N. (2012). Spray Drying Technique of Fruit Juice Powder: Some Factors Influencing the Properties of Product. Int. Food Res. J..

[46] Fernandes R.V.D.B., Borges S.V., Botrel D.A. (2014). Gum Arabic/Starch/Maltodextrin/Inulin as Wall Materials on the Microencapsulation of Rosemary Essential Oil. Carbohydr. Polym..

[47] Pudziuvelyte L., Marksa M., Jakstas V., Ivanauskas L., Kopustinskiene D.M., Bernatoniene J. (2019). Microencapsulation of Elsholtzia Ciliata Herb Ethanolic Extract by Spray-Drying: Impact of Resistant-Maltodextrin Complemented with Sodium Caseinate, Skim Milk, and Beta-Cyclodextrin on the Quality of Spray-Dried Powders. Molecules.

[48] Zokti J.A., Baharin B.S., Mohammed A.S., Abas F. (2016). Green Tea Leaves Extract: Microencapsulation, Physicochemical and Storage Stability Study. Molecules.

[49] Ali B.H., Ziada A., Blunden G. (2009). Biological Effects of Gum Arabic: A Review of Some Recent Research. Food Chem. Toxicol..

[50] Rodríguez-Hernández G.R., González-García R., Grajales-Lagunes A., Ruiz-Cabrera M.A., Abud-Archila M. (2005). Spray-Drying of Cactus Pear Juice (*Opuntia streptacantha*): Effect on the Physicochemical Properties of Powder and Reconstituted Product. Dry Technol..

[51] Tonon R.V., Freitas S.S., Hubinger M.D. (2011). Spray Drying of Açai (*Euterpe oleraceae* Mart.) Juice: Effect of Inlet Air Temperature and Type of Carrier Agent. J. Food Process. Preserv..

[52] Mishra P., Mishra S., Mahanta C.L. (2014). Effect of Maltodextrin Concentration and Inlet Temperature during Spray Drying on Physicochemical and Antioxidant Properties of Amla (*Emblica officinalis*) Juice Powder. Food Bioprod. Process..

[53] Zorić Z., Pelaić Z., Pedisić S., Elez Garofulić I., Bursać Kovačević D., Dragović–Uzelac V. (2017). Effect of Storage Conditions on Phenolic Content and Antioxidant Capacity of Spray Dried Sour Cherry Powder. Food Sci. Technol..

[54] Watson M.A., Lea J.M., Bett-Garber K.L. (2017). Spray Drying of Pomegranate Juice Using Maltodextrin/Cyclodextrin Blends as the Wall Material. Food Sci. Nutr..

[55] Chong P.H., Yusof Y.A., Aziz M.G., Nazli N. (2014). Mohd.; Chin, N.L.; Muhammad, S.K.S. Effects of Spray Drying Conditions of Microencapsulation of Amaranthus Gangeticus Extract on Drying Behaviour. Agric. Agric. Sci. Proc..

[56] Shahidi F., Peng H. (2018). Bioaccessibility and Bioavailability of Phenolic Compounds. J. Food Bioact..

[57] Ydjedd S., Bouriche S., López-Nicolás R., Sánchez-Moya T., Frontela-Saseta C., Ros-Berruezo G., Rezgui F., Louaileche H., Kati D.E. (2017). Effect of in Vitro Gastrointestinal Digestion on Encapsulated and Nonencapsulated Phenolic Compounds of Carob (*Ceratonia siliqua* L.) Pulp Extracts and Their Antioxidant Capacity. J. Agric. Food Chem..

[58] Tuan M.P., Hoang T.V.A., Le T.H.C., Vu N.Q.C., Dang T.B.O., Dang B.K., Pham D.T.M., Mai H.T., Nguyen T.T.S., Dam S.M. (2016). Extraction and Encapsulation of Polyphenols of Guava Leaves. Annals. Food Sci. Technol..

[59] Jovanović M., Drinić Z., Bigović D., Zdunić G., Mudrić J., Šavikin K. (2021). Effect of Carrier Type on the Spray-Dried Willowherb (*Epilobium angustifolium* L.) Leaves Extract, Powder Properties and Bioactive Compounds Encapsulation. Lek. Sirovine.

[60] Bonetti G., Tedeschi P., Meca G., Bertelli D., Mañes J., Brandolini V., Maietti A. (2016). In Vitro Bioaccessibility, Transepithelial Transport and Antioxidant Activity of *Urtica dioica* L. Phenolic Compounds in Nettle Based Food Products. Food Funct..

[61] Bhusari S.N., Kumar P. Antioxidant activities of spray dried tamarind pulp powder as affected by carrier type and their addition rate. Proceedings of the International Conference on Food, Biological and Medical Sciences (FBMS-2014).

[62] Fernandes M.R.V., Kabeya L.M., Souza C.R.F., Massarioli A.P., Alencar S.M., Oliveira W.P. (2018). Antioxidant Activity of Spray-Dried Extracts of Psidium Guajava Leaves. J. Food Res..

[63] Sharayei P., Azarpazhooh E., Ramaswamy H.S. (2020). Effect of Microencapsulation on Antioxidant and Antifungal Properties of Aqueous Extract of Pomegranate Peel. J. Food Sci. Technol..

[64] Pinho E., Grootveld M., Soares G., Henriques M. (2014). Cyclodextrins as Encapsulation Agents for Plant Bioactive Compounds. Carbohydr. Polym..

[65] Carvalho A.R., Costa G., Figueirinha A., Liberal J., Prior J.A.V., Lopes M.C., Cruz M.T., Batista M.T. (2017). *Urtica* Spp.: Phenolic Composition, Safety, Antioxidant and Anti-Inflammatory Activities. Food Res. Int..

[66] Pinelli P., Ieri F., Vignolini P., Bacci L., Baronti S., Romani A. (2008). Extraction and HPLC Analysis of Phenolic Compounds in Leaves, Stalks, and Textile Fibers of *Urtica dioica* L.. J. Agric. Food Chem..

