# Supplementary material for: Effect of Spray Drying Encapsulation on Nettle Leaf Extract Powder Properties, Polyphenols and Their Bioavailability
Source: Foods. 2022 Sep 15;11(18):2852. doi: 10.3390/foods11182852 (PMC9498331; doi:10.3390/foods11182852)
Supplement: Supplementary file 1 [file foods-11-02852-s001.zip › foods-1893829-supplementary/Figure S1.pdf]

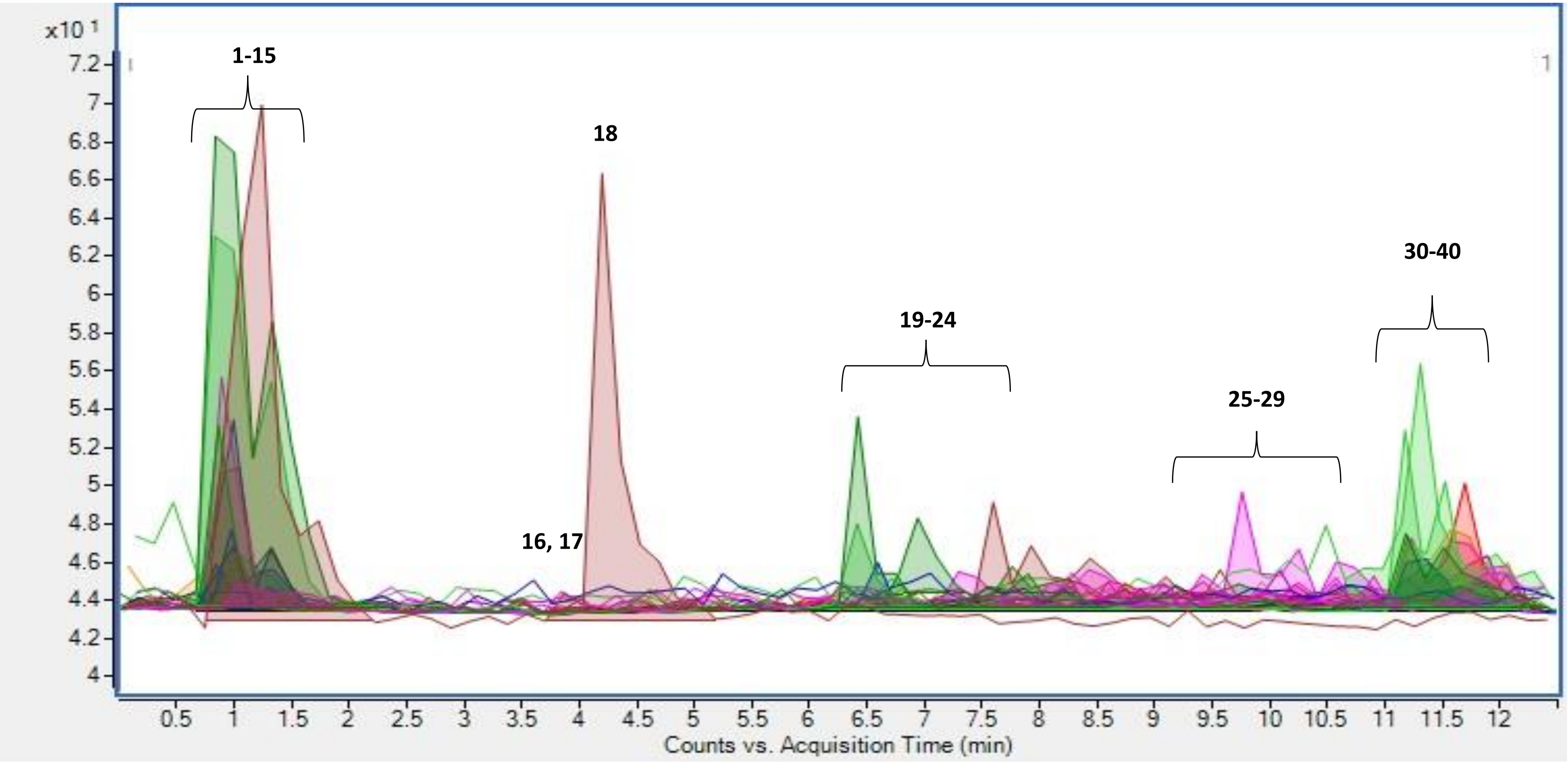

Figure S1. UPLC-MS/MS chromatogram in MRM acquisition mode from nettle leaf encapsulated extract obtained at 160 °C with β-CD:GA (3:1) and sample:carrier ratio 1:3. Peak designation was: (1) Quinic acid (2) Umbelliferone (3) Protocatechuic acid (4) Chlorogenic acid (5) Scopoletin (6) Naringenin (7) Genistic acid (8) Myricetin (9) Luteolin (10) Isorhamnetin 3-O-rutinoside (11) Caffeic acid (12) Esculetin (13) Apigenin 7-O-glucoside (14) Kaempferol 3-O-rutinoside (15) Kaempferol 3-O-glucoside (16) *p*-coumaric acid (17) Sinapic acid (18) Cinnamic acid (19) Isorhamnetin (20) Ferulic acid (21) Apigenin (22) Genistein (23) Quercetin (24) Kaempferol pentoside (25) Quercetin pentoside (26) Epigallocatechin gallate (27) Syringic acid (28) Kaempferol rhamnoside (29) Epicatechin gallate (30) Catechin (31) Quercetin 3-O-rutinoside (32) Gallic acid (33) *p*-hydroxybenzoic acid (34) Quercetin-acetylrutinoside (35) Kaempferol pentosyl-hexoside (36) Quercetin pentosyl-hexoside (37) Quercetin acetyl-hexoside (38) Kaempferol (39) Quercetin 3-O-rhamnoside (40) Epicatehin
